# Immunogenetics and TCR expression patterns together predict future T1D onset from multi-omic datasets

**DOI:** 10.3389/fgene.2026.1833788

**Published:** 2026-07-30

**Authors:** Lufan Yang, Alexis Lindahl, Michael Robben

**Affiliations:** 1 Department of Animal Science, University of Illinois, Urbana-Champaign, Urbana, IL, United States; 2 Division of Nutritional Science, University of Illinois, Urbana-Champaign, Urbana, IL, United States; 3 Institute for Genomic Biology, University of Illinois, Urbana-Champaign, Urbana, IL, United States

**Keywords:** HLA, RNA-seq, ScRNA-seq, statistical modeling, T cell receptor

## Abstract

Type 1 Diabetes (T1D) is an autoimmune disease affecting juvenile populations across the world. Progression to symptomatic disease, currently predicted by ancestral risk and the presence of islet reactive autoantibodies (AAB), is prone to costly diagnostic error. The relative importance of T cells to disease progression have long made it a target for prognostic markers, but the complexity of T cell sequencing data has hindered it’s application in the field. In this study, we investigated the multiple types of TCR sequencing data as a source of predictive features for future onset of T1D. Using a large dataset of over 730 TCR repertoires from 321 patients, we found that TCR gene usage was highly predictive for clinical disease risk. Immunogenetic background played an important role in feature selection, stressing the importance of personalized medicine approaches to prognostic marker identification. We demonstrate that the same predictive features can be extracted from bulk RNA-seq data which is cheaper to measure than TCR profiling data. The features identified in this study can be used to measure disease progression in pre-clinical samples opening the door for future development of fast and accessible blood tests for T1D.

## 1 Introduction

Type 1 Diabetes is an autoimmune condition affecting 3 million individuals worldwide (Katsarou et al., 2017). Symptomatic disease most commonly occurs in juvenile populations under the age of 14 but requires chronic treatment throughout life. The cause of T1D is T cell mediated immune responses to pancreatic antigens leading to autoimmune destruction of pancreatic islet β cells. The disease has no known cure and the current best treatment is preventative therapy administered before clinical stage 3 presentation (Maines et al., 2025).

T1D is currently detected through seropositivity of auto antibodies (AAB) specific to islet antigens such as Insulin autoantigen (IAA), glutamic acid decarboxylase (GADA), zinc transporter 8 (Znt8A) and insulinoma associated antigen (IA-2A) (Ziegler et al., 2013). Detection of multiple AAB before the age of 20 has a 88% accuracy in predicting future disease course, however not all patients develop multiple AAB or maintain both AAB prior to disease onset (Frohnert et al., 2023). Single AAB detection by IAA or GADA represents >70% of screened patients yet false positives (type I error) range between 50%–60% (Yu et al., 2017). Rate of false negatives (type II error) in individuals with no AAB detection can be as high as 20%.

AAB are commonly only detectable within a specific time window making it impractical to screen every child, so at the moment the standard criteria is children with a first degree relative with symptomatic disease (Hussain et al., 2026). However an astonishing 85% of newly diagnosed cases have no first degree relative, leaving the vast majority of future T1D patients without detection until the sudden onset of symptoms (Rewers and Ludvigsson, 2016). While a clear genetic cause has not been found for this disease, genetic risk factors have been shown to be highly correlative with disease (Sharp et al., 2019; McGrail et al., 2026). Symptomatic disease progression is heavily affected by lifestyle and environmental factors which are not subject to genetic background (Redondo et al., 2018).

Human studies have characterized increased infiltration of autoantigen specific CD8^+^ T cells into the pancreas prior to disease onset (Coppieters et al., 2012). Yet, unlike B cells, T cells have received less attention as potential markers of disease. One reason is that a B cell assay is easier to implement, as B cell targets can be detected with a single source protein, whereas T cell epitopes are numerous and are restricted to different Major Histocompatability Complex (MHC) alleles that vary in each individual (Lee and Meyerson, 2021). Another reason is that auto-reactive T cells comprise only a small fraction of circulating T lymphocytes, and their response to self-peptides is characteristically weak (Unger et al., 2012; Ahmed et al., 2019). Some research has focused on asking the question if the T cell and it’s T cell receptor (TCR) can act as a fingerprint of immune status that can be used to detect disease before symptoms occur (Pauken et al., 2022). One group found signatures in the CDR3 region that associates specifically with epitopes for rheumatoid arthritis (Ishigaki et al., 2022).

Previous studies have conducted TCRα and TCRβ profiling in individuals with T1D to understand the effect of diabetes on the T cell repertoire (Kent et al., 2005; Seay et al., 2016; Gomez-Tourino et al., 2017; Jacobsen et al., 2017; Anderson et al., 2021; Mitchell et al., 2023; Hanna et al., 2025). While the studies recognize common changes to the repertoire, they do not isolate consistent TCR features that may be predictive of disease. Other approaches to T cell analysis using bulk RNA-seq (Dufort et al., 2019; Carry et al., 2022; Codazzi et al., 2025; Valentim, 2025) and scRNA/scTCR-seq (Cerosaletti et al., 2017; Okamura et al., 2022; Eugster et al., 2024; Honardoost et al., 2024; Abdullatypov et al., 2025) have contributed to our understanding of how T cells change during diabetic development in humans to potentially identify TCR independent markers of disease. We previously performed single cell RNA and TCR-sequencing in the NOD mouse model of T1D and found that T cells that infiltrate the pancreas express specific TCR diversity genes (V, D, and J segments) at higher rates than expected (Islam et al., 2025). In this study, we sought to unify information from different kinds of T cell sequencing datasets to determine if there are universal features that can predict future disease onset in individuals. Based on differential analysis and statistical and machine learning models, we identify many potential T cell sequence markers of disease risk that are commonly shared and highlight the dependence on immunogenetics in making predictions.

## 2 Methods

### 2.1 TCR profiling data

In total, 993 TCR profiling files in standard AIRR format from 7 TCRαβ sequencing studies were downloaded from the IReceptor database (Corrie et al., 2018; Hanna et al., 2025). TCR profiling files for each sample were used to create a single matrix containing the number of reads per sample detected for each unique clone (CDR3 abundance). Unique CDR3 were filtered to remove clones with low read counts (<10) across all samples. CDR3 sequences less than 6 amino acids (AA) were also removed to correct for sequencing and annotation error. Samples with low coverage (less than 10,000 unique CDR3 detected were removed to reduce batch effects. CDR3 usage in case and control patients were calculated from the proportion of samples with positive detection of unique CDR3 (abundance >0) to the total number of samples in that disease status.

Because disease status was reported differently in each study, the definition was normalized between studies. Cases in which the data was derived from patients were symptomatic for stage 2 or 3 diabetes (fasting OGTT >6 mg/dL, HbA1c > 39 mmol/L, or ≥2 AAB detected) were classified as “case”. Stage 1 diabetic, single AAB positive juveniles, and healthy controls were classified as “control”. In instances where multiple time points were available, the last available time points were used for disease classification. To evaluate pre-diabetic risk, timing of measurement was classified per sample based on if the sample was taken at the terminal point of study measurement (last existing record for patient).

Patients were evaluated for genetic background of Human Leukocyte Antigen (HLA) alleles based on available reports. Patients with 0 reported genetic alleles were removed from the dataset. On average, each patient was genotyped for 4 Major Histocompatibility Complex (MHC) class I and II alleles. Genotyped alleles were classified as “high-risk” or “highly-protective” based on previous reports of T1D genetic risk factors (Noble and Valdes, 2011; Hadley et al., 2015; Onengut-Gumuscu et al., 2019; Jiang et al., 2021; Liu et al., 2021; Al Yafei et al., 2022; Besançon et al., 2022; Dashti et al., 2023). Patients were classified as “At-risk” if they carried only a high risk allele, “Protected” if they carried only a protective allele, “Both” if they carried one of each, and “Neither” if they carried neither risk nor protective alleles.

### 2.2 CDR3 annotation and aggregation

Exact CDR3 matches were annotated to experimentally validated epitope targets using the VDJdb (downloaded 25 Feb 2026) (Shugay et al., 2018). Unique CDR3 were grouped by the targeted epitope source gene as opposed to epitopes to maximize coverage of antigen specific CDR3 across samples. Antigen specific clonal abundance was aggregated from the sum per sample of the unique CDR3 targeting the same source protein. Antigen proteins were annotated as “Human” or “Non-human” if the source organism of the epitope was labeled “*Homo sapiens*” or not, and “Human T1D” if they matched known epitope targets of T1D (Roep and Peakman, 2012). Antigen specific clone abundance was normalized across samples by total abundance in each sample.

CDR3 sequences were clustered using the GIANA (Zhang et al., 2021) clustering algorithm. Coverage was calculated as the CDR3 usage in total patients per number of CDR3 in each cluster. Clusters with less than 4 CDR3 were removed because they were found in less than 70% of patients on average. Cluster abundance was then calculated from the sum aggregate of abundance from all CDR3 in a cluster per sample. Cluster abundance was normalized to the number of unique CDR3 per cluster before being normalized by total abundance in each sample.

The TCR gene identity of each unique CDR3 was determined from the AIRR outputs of the original sample files. TCR gene abundance was then predicted by summing the clonal abundance of CDR3 sequences using specific TCRα and TCRβ V, D, and J genes. Abundance was then normalized to the total number of reads detected per individual based on the sum of reads from CDR3 that were antigen-specific, cluster-specific, or TCR gene-specific.

### 2.3 Statistical analysis of TCR profiling data

Shannon diversity was calculated using the vegan package in R for each patient. Wilcoxon-ranked sum tests were performed to compare the distribution Shannon indexes of case vs. control samples for each combination of terminal measurement status and allele risk level. A random sample of 100 TCR profiling abundances were used to calculate pairwise rarefication using the vegan package in R. Bray-Curtis distance was then calculated from rarified samples also using the vegan package.

Enrichment of clones was calculated using a fisher’s exact test, to determine if there is a higher probability of finding the clone than expected. Significance was adjusted by the False Discovery Rate (FDR) to account for random chance in large numbers of CDR3. Changes in abundance were evaluated by calculating the meaning of normalized among patients in case and control. Differential abundance of overrepresented CDR3, specific antigen clones, clonal clusters and TCR genes was determined from FDR adjusted p-values using Welch’s t-test. The means of subsets of case and control samples were used to evaluate the effects of timing of measurement and allelic risk level.

### 2.4 Statistical modeling

TCR gene abundance was modeled for its effect on predicting future disease status using logistic regression. A full feature dataset was assembled with samples as rows and each feature as a separate column. Phenotypic features always used included age, sex, and study country as variables. Normalized and scaled TCR abundance or expression for every TCR gene to values between 0 and 1 were included as TCR features. An identity matrix with the per patient recorded HLA alleles represented as 1 for detected and 0 for not detected was included as HLA features. Unless otherwise specified, models were trained on a 70–30 train-test split balanced for equal representation of case and control samples.

Statistical models that could account for each patient’s genetic backgrounds were fit to normalized TCR gene usage. Accuracy and R^2^ were predicted for each allele to determine genotype specific fit to each model. Accuracy was calculated from the predicted probabilities of the regressor by using a threshold value of 0.5. R^2^ in logistic regression was calculated using Tjurk’s coefficient of determination with the formula:

$$R^{2}=mean\left(p_{pass}\right)-mean\left(p_{fail}\right)$$

Where *p*_*pass*_ is the vector of probabilities for cases with diabetes and *p*_*fail*_ is the vector of probabilities for healthy controls. False positive (FPR, type I error) and false negative (FNR, type II error) rates were calculated as:

$$FPR=\frac{FP}{FP+TN}$$

$$\;FNR=\frac{FN}{FN+TP}$$

Where TN and TP are the number of true negatives and true positives. AUC of predictions was calculated using the pROC package in R (Robin et al., 2011). Polygenic models were developed by training models on patients who share two specific alleles and were only developed for subsets of allele combinations with at least one case and control sample.

#### 2.4.1 Generalized linear model

The generalized linear model (GLM) was fitted using the cv. glmnet function from the glmnet package in R, with a “binomial” family link function (Friedman et al., 2010). The GLM was trained using Age, HLA, and TCR gene usage (Full), only age and HLA features (HLA only), or only age and TCR gene features (TCR only), resulting in three different models. The HLA only model resulted in perfect prediction due to high multicollinearity in the dataset but was not adjusted to reduce overfitting.

#### 2.4.2 Multivariate model

A multivariate (MV) model was trained to predict the binomial dependent variable for each allelic identity (i.e., For each of the k detected MHC alleles there is a unique model predicting the logistic diabetic status), using the following formula:

$$f\left(x_{k}\right)=\beta_{0k}+\sum \beta_{mk}*x_{m}+\in$$

Where *β*_*0k*_ is the *k* ^*th*^ intercept and *β*_*tk*_ is the coefficient of the *m* ^*th*^ TCR gene for the *k* ^*th*^ model and *x*_*t*_ is the normalized clonality of the *m* ^*th*^ TCR gene. Overall patient accuracy was calculated as the mean of accuracy for each individual *k* model. Coefficients of each TCR gene was calculated as the mean of coefficients for each model. We observed equal variance for coefficients from each model. The intercept of each model represented the contribution from each allele.

#### 2.4.3 Linear mixed model

A linear mixed model (LMM) was developed using random intercepts to model the effect of genotype. TCR gene usage was used as predictors of binomial disease status using the formula:

$$f\left(x\right)=X_{mxn}\beta_{m}+Z_{kxn}u_{k}+\in$$

In this model, *X* represents the *m x n* feature matrix of the *m* TCR genes for each *n* sample. It is multiplied by the *m* length matrix of coefficients, one for each TCR gene. *Z* represents a *k x n* identity matrix of *k* HLA alleles for each *n* sample. We use this array to select a random intercept from the *k* dimensional *u* array, resulting in a predictor calculated for each allele for each patient. Accuracy was then calculated for each allele from the mean of the probabilities of each patient with that allele. R^2^ for each allele was also calculated using these mean probabilities for all patients with that allele. This model thus controls for the random effect of genotype by adding a different intercept for each allele.

#### 2.4.4 Machine learning

Machine learning models were developed from feature datasets including only TCR features (TCR only) or TCR and HLA features (TCR+HLA) using the Caret package in R (Kuhn, 2008). The models used were Random Forest with bagging (Bag), Sparse Discriminant Analysis (SDA), Multilayer Perceptron (MLP), Generalized Linear Model (GLM), Gradient boost (Boost), and Support Vector Machine (SVM). Models were trained on a 70–30 train test split using a 10 fold cross validation with hyper parameterization of individual models. Accuracy and AUC were calculated as previously stated for each cross fold, resulting in multiple accuracies for the same model trained on different portions of the data but tested on the same portion. Feature selection was performed using recursive feature elimination where random forests were developed from different subsets of features to identify which features and how many are required to achieve a specific level of accuracy.

### 2.5 RNA seq file processing

Single and paired end fastq files were downloaded from the NIH SRA database (Dufort et al., 2019; Carry et al., 2022; Codazzi et al., 2025; Valentim, 2025). Phenotypic data was obtained from each study for patient each patient and normalized to be comparable between studies. Cases in which data was derived from patients were symptomatic for stage 2 or 3 diabetes (fasting OGTT >6 mg/dL, HbA1c > 39 mmol/L, or ≥2 AAB detected) were classified as “case”. Stage 1 diabetic, single AAB positive juveniles, and healthy controls were classified as “control”. DAISY samples reported some patients as “reverters”, who were once AAB positive (no reported number of AAB) but have become no longer AAB and were considered as older control patients. Instead of using terminal measurements due to the variation in age ranges among studies, samples were considered “Terminal” if they were taken at an age greater than 12, where we observed the biggest change in TCR expression data. Code was written to accommodate both single and paired end sequencing files through the same pipeline and no major steps were changed based on file type. Files were trimmed to an average quality of 24 PHRED score before alignment.

### 2.6 RNA seq alignment and CDR3 annotation

All files were aligned to the human genome (GRCH38. p14) using the STAR aligner with default settings (Dobin et al., 2013) reaching an average alignment of 83% of reads. The HTseq program was used to count read alignment to gene transcripts from the GTF file (Putri et al., 2022). Transcript quantification was required in order to quantify TCR V, D, J, and C gene segments but did not adversely affect gene quantification. Trimmed reads were used as input to the MIXCR program using the ‘analyze rna-seq’ preset, which output predicted CDR3 and gene segments for TCRα, TCRβ, TCRγ and TCRδ genes in AIRR format.

### 2.7 Allele prediction and risk evaluation

Trimmed reads were run through the T1K program to predict major alleles of MHC class I and II, MIC and TAP genes (Song et al., 2023). Alleles were aligned to the reference IPD-IMGT/HLA v3.44.0 database which contains 46,000 allelic variants. The allelic identity matrix was calculated for all 1,142 detected alleles for each sample. Sample completeness was calculated as the number of heterozygous alleles from 26 major loci detected. Prediction accuracy was calculated based on error rate in allele calls from samples of the same patient. OR of each allele was calculated as:

OR=CaseeControleCasenControln

where *e* represents case and control patients exposed to the allele and *n* represents case and control patients not exposed to the allele.

### 2.8 Expression, TCR gene analysis, and allele importance

Count read files were imported into R and assembled into a count matrix using custom scripts. General expression differences were derived from differential expression analysis of disease affected individuals across studies using the DESeq2 and clusterProfiler package (Yu et al., 2012; Love et al., 2014). TCR genes were extracted from the count matrix and normalized across studies using the SCT transformation (Choudhary and Satija, 2022). Differential expression of TCR genes was calculated per gene using the expected values from the negative binomial derived from the SCT. Patients were categorized as containing a high-risk or highly-protective allele, both or neither as before.

### 2.9 Single cell TCR gene analysis

Human 3’ single-cell RNA-seq (scRNA-seq) count data from the spleen and lymph node (LN) of diabetic patients and healthy donors (Fasolino et al., 2022; Patil et al., 2023) was downloaded from the Panc-DB database as preprocessed H5ad files (Kaestner et al., 2019; Fasolino et al., 2022). Per patient metadata was matched to cells from the same individual and included clinical disease status, age, sex, BMI, HbA1C, C-Peptide, Class I and II MHC alleles, and positivity of GADA, IA-2, IAA, and ZnT8 AAB. Cells from T2D patients with no AAB positivity were removed from the dataset. Patients were then classified as healthy (“HD”) if they had no clinical symptoms or AAB detected, “AAB+” if they had no clinical symptoms but a single AAB detected, and “T1D” if they had clinical symptoms and/or multiple positive AAB tests.

Cells were further filtered by quality metrics to retain cells with mitochondrial transcript fractions less than 20% and nFeatures >200 and <2,500. T cells were identified and subsetted based on CD3D/CD3E expression using Seurat (v 5.3.0) (Hao et al., 2024). Raw RNA counts were normalized with SCTransform (Choudhary and Satija, 2022) and batch correction was performed using Harmony (Korsunsky et al., 2019). Principal components from the top 2000 most variable genes were used to produce Leiden clusters at multiple resolutions. The resolution that produced the most stable clusters with the least leakage of cells between clusters (10 total clusters) were further used to predict markers with the Seurat FindAllMarkers function. Markers were compared to reference T cell subset genes to annotate T cell clusters (Szabo et al., 2019). Cell proportion was calculated per patient from total leukocyte population.

TCR genes were identified based on previous analysis of bulk RNA-seq data. Cells were classified per TCR gene based on detection of >1 raw reads. Usage was determined based on the proportion of positive TCR genes to total leukocytes in each patient. Cells with single positivity for one TCR gene were annotated as TCR gene containing clusters (few cells had detected multiple TCR genes even though it was possible). Multiple variable non-parametric Kruskal-Wallis tests were performed on the TCR gene usage for each TCR gene cluster for different disease status, # of AAB, and cell type, followed by *post hoc* wilcoxon ranked sum tests. Differential gene expression for each TCR gene cluster was determined using the FindMarkers function in the comparisons between T1D vs. HD and AAB vs. HD. Statistical p-values were corrected using the Benjamini-Hochberg (BH) method. Functional enrichment of GO terms was determined by Gene Set Enrichment Analysis (GSEA) using the clusterProfiler package (Yu et al., 2012).

### 2.10 Mouse dPCR

NOD/ShiLtJ mouse blood samples that were previously collected (Islam et al., 2025) at 6, 14, and 24 weeks of age were used as a source for TCR gene RNA. Viably frozen PBMCs were washed to remove DMSO. Each sample was seeded in a well of a 96-round bottom plate in 200 µL of culture medium with 30 U/mL IL-2 and a 1:1 concentration of cells to αCD3/CD28 Mouse Dynabeads (Gibco, #11456D). Cells were expanded to a count of 1 × 10^6^ total cells and resuspended in 500 µL TRIzol (Invitrogen, #15-596-026). RNA was extracted by chloroform-isopropanol precipitation on ice and washed twice with 75% ethanol. Pellets were dried at 25 °C before being resuspended in nuclease-free water and measured for quality and quantity by nanodrop. Approximately 50 ng of RNA from each underwent first strand cDNA synthesis with 6 µM poly dT and 200 U M-MuLV Reverse Transcriptase (New England Biolabs, M0253S). Primers for trav16n (F: 5′-GTA​CAA​GCA​AAC​AGC​AAG​TG-3′), trav7 (F: 5′-AGAAGGTRCAGCAGAGCCCAGAATC-3′), trbv29 (F: 5′- GCT​GGA​ATG​TGG​ACA​GG-3′) and trbv14 (F: 5′-GCA​GTC​CTA​CAG​GAA​GGG-3′) were used for dPCR quantification of copy number from cDNA samples, with the reverse primer for TCRα (R: 5′-GGC​ATC​ACA​GGG​AAC​G-3′) and TCRβ (R: 5′-CCA​GAA​GGT​AGC​AGA​GAC​CC-3′) depending on chain. EvaGreen dPCR reactions were prepared according to the 2x master mix kit (Qiagen, #25011) for each sample and primer combination. A total of 1 µL of undiluted cDNA was used to calculate total TCR copy number based on dilution series performed before tests. Reactions were run for 95 °C for 2 min, followed by 35 cycles of: 95 °C for 30 s, 55 °C for 30 s, and 72 °C for 30 s, and a final extension of 72 °C for 30 s then imaged on the green channel. The Quiacuity software was used to calculate the total number of copies/µL in the original undiluted sample. Samples were normalized to expression of the housekeeping gene B2M (F:5′- CCT​GTA​TGC​TAT​CCA​GAA​AAC​CCC-3’; R: 5′-GCA​GTT​CAG​TAT​GTT​CGG​CTT-3′) using the formula:

$$Normalized\;expression=\frac{n_{copies}}{n_{housekeeping}}$$

Where *n* is the raw output of the dPCR for each TCR gene or B2M of that sample in copies/µL, resulting in the copies of TCR gene per copy of B2M. Mean normalized expression of case and control samples at each time point were used as parameters of a Student’s t-test.

### 2.11 Code and data availability

TCR profiling data used in this study was downloaded from the IReceptor database (www.IReceptor.org, Downloaded 14 June 2025) (Supplementary Table S1). Bulk RNA-seq data was downloaded as fastq files from the SRA database from the datasets: GSE124400, GSE287275, GSE230370, and GSE123658 (Downloaded 5 January 2026) (Supplementary Table S8). Single cell RNA-seq data from the spleen and lymph nodes of donor patients were downloaded from Panc-DB as H5ad files (Downloaded 2 Feb 2026). Code to reproduce the analysis is available in the project GitHub page: https://github.com/RobbenLab/T1D_TCR_Meta.

## 3 Results

### 3.1 Evaluation of CDR3 sequencing data for determining diabetic risk

**FIGURE 1:**
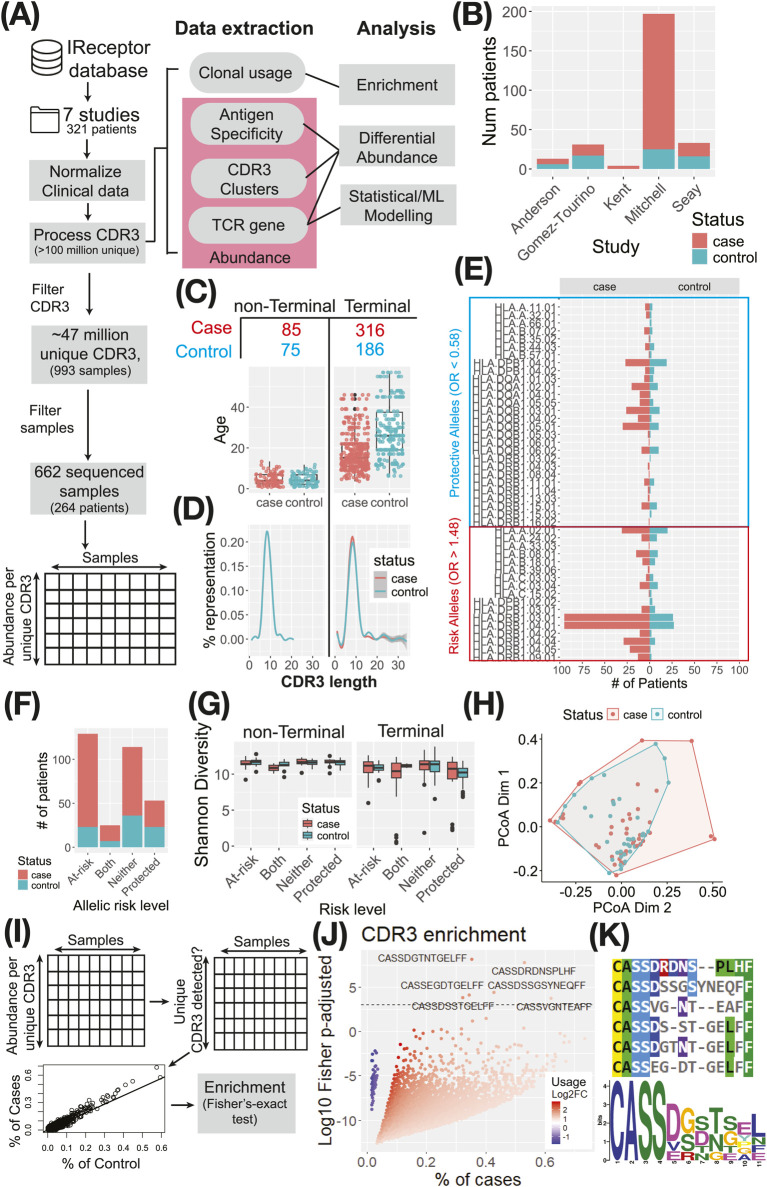
Characteristics of TCR profiling data from diabetic and non-diabetic populations. **(A)** PRISMA diagram showing data selection and curation. CDR3 data from 7 different studies (321 patients, 993 samples) was downloaded from the IReceptor database. Low quality unique CDR3 with average read coverage, less than 10 were filtered out. Samples with fewer than 10,000 unique clones were detected prior to downstream analysis. Per sample CDR3 abundance was determined for each unique CDR3 from the number of reads detected in each patient. **(B)** The number of patients across 5 studies varied based on disease status. **(C)** Patients were classified as “case” if they had or were highly likely to reach stage 3 clinical disease status based on data reported by each study. Samples were classified as “terminal” if they matched the last reported age of patients. Terminal samples had a higher median age than non-terminal samples (bottom). **(D)** The cumulative abundance of different length CDR3 was calculated per patient and then divided by total abundance in “case” or “control” samples. **(E)** The presence of risk or protective alleles was determined in each individual using results from previous studies. The number of patients with positive detection was plotted for each risk (red box) and protective (blue box) allele. **(F)** Patients were classified by allelic risk based on the detection of risk or protective alleles and the number of patients in each classification are represented in a bar chart. **(G)** Shannon diversity was calculated for each individual by allelic risk factor and terminal measurement status. **(H)** Beta diversity was calculated using rarified bray-curtis distance and plotted using a Principal Coordinate Analysis (PCoA). **(I)** Each unique CDR3 was tested to determine if it was enriched in case samples at a higher rate than expected. The scatter plot in the bottom right shows CDR3 usage (proportion of samples detected in) for case vs. control samples. The line of best fit (black line) shows an overall linear trend in CDR3 usage. **(J)** The -Log10 adjusted p-value of clones that had a >1 log2 fold change difference in CDR3 usage were plotted by proportion of case samples and colored by log2 fold change. The horizontal line represents an alpha of 0.05 in the FDR adjusted p-value from a fisher’s-exact test. Points were labeled if they had an adjusted p-value less than 0.05. **(K)** Local alignment of the 6 significantly enriched CDR3 sequences using a clustal-w algorithm (bottom). A position weight matrix shows the relative sequence frequency of the aligned sequences (top).

To evaluate if T-cell sequencing and immunogenetic data can be predictive of future T1D onset we performed meta-analysis on publicly available TCR profiling data from 7 different studies (Figure 1A). In total, 993 TCRα or TCRβ sequencing files representing 321 patients and 117,618,904 unique clones were compiled for use in this study. No differences in sex or race were observed between studies (Supplementary Table S2). Age varied per study and 55% of patients had multiple sequencing files sampled from different ages, tissues, or cell types (Supplementary Figure S1). Patients were classified for disease status as “case” if they presented for stage 2 or 3 clinical disease based on hba1c, c-peptide, blood glucose, or multiple AAB detection (Figure 1B). To capture the timing of disease measurements, samples were classified as “Terminal” or “Non-terminal”. Terminal samples presented at later median age than non-terminal samples, with 80% of samples taken after age 14, the peak juvenile diagnostic age (Leete et al., 2018) (Figure 1C).

Unique CDR3 sequences were filtered to remove lowly detected reads (<10 reads across all patients). We found that CDR3 length did not differ based on timing of measurement or disease status (Figure 1D). CDR3 shorter than 6 amino acids (AA) were removed as likely sequencing errors. We ended up with a total of 47,994,372 TCRα and TCRβ CDR3 sequences which were compiled into a sparse abundance matrix. To reduce variability between studies, samples with less than 10,000 unique CDR3 detected were removed, resulting in 662 samples representing 264 patients.

Genotype information varied per study with an average of 7 MHC class I and II alleles classified per patient. We observed a slight bias towards MHC class II alleles across studies (Supplementary Figure S2), likely to focus on characterization of the high-risk DR3/4 alleles. Based on all known previous characterizations of HLA allelic risk for T1D onset (Supplementary Table S3), we observed 179 patients that contained at least one highly-protective allele (OR > 1.48, 61 case and 118 control) and 217 patients that contained at least one high-risk allele (OR < 0.58, 182 case and 35 control) (Figure 1E). We classified patients by allelic risk levels if they had a high-risk allele (“At-risk”), a protective allele (“Protected”), “Both” alleles, or “Neither” (Figure 1F).

The average number of unique CDR3 sequences detected per individual was 108,967 but varied due to tissue source and sequencing depth (Supplementary Figures S3A,B). Only 9% of clones could be found in more than 2 patients and any two individuals shared less than 1% of clones between them on average (Supplementary Figures S3C,D). We found very little difference in Shannon diversity between case and control samples even when stratified by measurement timing and allelic risk (Figure 1G). This was consistent with measurements of beta-diversity (Figure 1H). Our results on CDR3 length and diversity in diseased populations are inconsistent with conclusions from other TCR profiling analyses which could be due to population diversity in samples (Gomez-Tourino et al., 2017; Rawat et al., 2026).

The relative proportion of patients containing any reads of a unique CDR3 (CDR3 usage) was used to calculate if any clones were specifically enriched in individuals with T1D (Figure 1I). Fishers exact tests on the observed proportion of cases compared to the expected found only 6 unique CDR3 sequences significantly enriched in case samples (“CASSDGTNTGELFF”, “CASSDRDNSPLHF”, “CASSDSSGSYNEQFF”, “CASSDSSTGELFF”, “CASSEGDTGELFF”, “CASSVGNTEAFF”) (Figure 1J). One of these sequences was found in a TCRβ clone specific for psoriasis vulgaris (Jirouš Drulak et al., 2023) and 2 others found in clones targeting SARS COV-2 targets (Snyder et al., 2024). These sequences shared little overall sequence identity at variable residues (Figure 1K) but overlapped for TCR genes TRBV7-3, TRBV11-2, and TRBV9-2 which are common in T1D specific clones (Nakayama and Michels, 2021).

### 3.2 Evaluation of antigen specificity, CDR3 clustering, and TCR gene usage for determining diabetic risk

**FIGURE 2:**
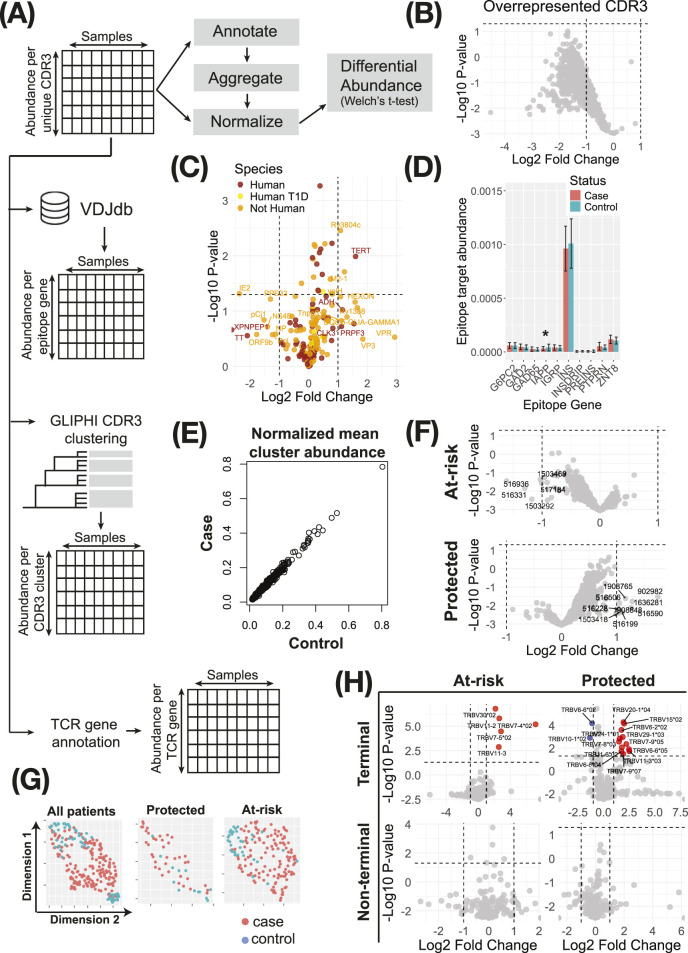
Comparison of TCR abundance in overrepresented CDR3 or groups of CDR3. **(A)** Average abundance between case and control samples values were compared using Welch’s t-test. **(B)** A volcano plot shows the log2 fold change of unique CDR3’s that were found in more than 50% of case and control patients. The horizontal line marks an FDR adjusted significant threshold of 0.05 and the vertical lines show a log2 fold change threshold of 1. Exact matches were used to find antigen specific CDR3 sequences in the VDJdb database and the cumulative abundance for each epitope gene was compared between diabetic and non-diabetic samples. **(C)** The differential abundance of antigen specific CDR3 sequences were plotted in a volcano plot. Species and disease source of each antigen is denoted by color. **(D)** Mean and standard deviation of cumulative abundance for each T1D relevant epitope target. *p < 0.05. GIANA clustering grouped CDR3 by sequence similarity. **(E)** Normalized mean cluster abundance showed little differentiation between diabetic and non-diabetic samples. **(F)** Volcano plots differential abundance of GIANA clusters based on allele risk level. Clusters with >1 Log2FC are labeled by cluster ID. Cumulative abundance per TCR gene was calculated for each patient. **(G)** Normalized TCR expression was reduced to two dimensions per patient using the UMAP algorithm and disease status is denoted by color (bottom). **(H)** Volcano plots show the number of differentially abundant TCR genes found in individuals with T1D broken down by terminal measurement status allele risk level.

Changes in relative abundance between case and control samples may differentiate risk of developing disease (Figure 2A). We found that 978 unique CDR3 were found in at least half of case and control samples across studies. We differentially compared the abundance of only unique CDR3 represented this way to reduce sampling error. None of the clones identified using these standards were significantly differentially abundant to an FDR adjusted p-value of 0.05 but most had a more than 2 fold reduction in abundance compared to control (Figure 2B). In terminal samples, we did observe 3 CDR3 upregulated in at-risk and 7 CDR3 upregulated in protected patients (Supplementary Table S4). None of these clones matched with those found significantly enriched (Figure 1J).

Unique CDR3 sequences were matched exactly to experimentally validated antigen specific CDR3 in the VDJdb. Only 3,290,000 (0.07%) of unique CDR3 matched to known antigen specific CDR3. Of the 178 epitope source genes that were identified as targets, 58% were of non-human source, 36% were of human source, and 6% were of human source and related to T1D development (Roep and Peakman, 2012). These targets included insulin (INS), pre-insulin (PRE-INS), Glutamic Acid Decarboxylase 2 (GAD2), GAD65, Glucose-6-Phosphate C2 (G6PC2), Islet Amyloid Precursor Protein (IAPP), Zinc Transporter 8 (ZNT8), and Protein Tyrosine Phosphatase Receptor Type N (PTPRN). We observed no differential abundance of the T1D related targets to a log2 fold change greater than 1 (Figure 2C; Supplementary Table S5). IAPP-targeting TCR abundance was significantly increased in case patients to an adjusted p-value <0.05 but we observed very little change in mean abundance between case and control (Figure 2D).

Clustering CDR3 clones by sequence similarity can group CDR3 by similar function (Feng et al., 2025). GIANA clustering resulted in the prediction of 2,018,873 clusters from 36,030,580 unique CDR3. The average cluster was made up of 17 unique CDR3 sequences and 73% of patients were detected per cluster when cluster size reached at least 3,000 CDR3 per cluster (Supplementary Figure S4A). With this cutoff, we ended up with 1,149 clusters which we used to calculate the normalized per cluster abundance (Supplementary Figure S4B). Normalized cluster abundance was highly linear between case and control patients (Figure 2E). Because of this, we did not observe any significant differentially abundant clusters, however when compared at different allelic risk levels, clusters showed opposite trends in log2 fold change (Figure 2F; Supplementary Table S6).

It is well supported that TCR clones that target the same antigen often use the same TCR gene (Gold, 1994). This led us to ask if abundance of CDR3 containing specific TCR genes would be consistent with disease risk. We detected 312 TCR V, D, and J genes from unique clones and calculated normalized abundance for each patient (Supplementary Figure S4C). Normalized TCR gene abundance differentiated case from control samples based on allelic risk level (Figure 2G). In diabetic samples, we identified 4 and 13 differentially abundant TCR genes in the terminal measurements of at-risk and protected patients respectively (Figure 2H; Supplementary Table S7). TRBV11-3 was commonly upregulated across allele risk levels (Supplementary Figures S4D,E) and associated with many T1D specific TCR clones (Nakayama and Michels, 2021). Altogether, TCR gene abundance seemed to be the most correlative feature with diabetes risk but was influenced by both timing of measurement and allelic risk level.

### 3.3 TCR gene abundance and immunogenetics are highly predictive of future disease status

**FIGURE 3:**
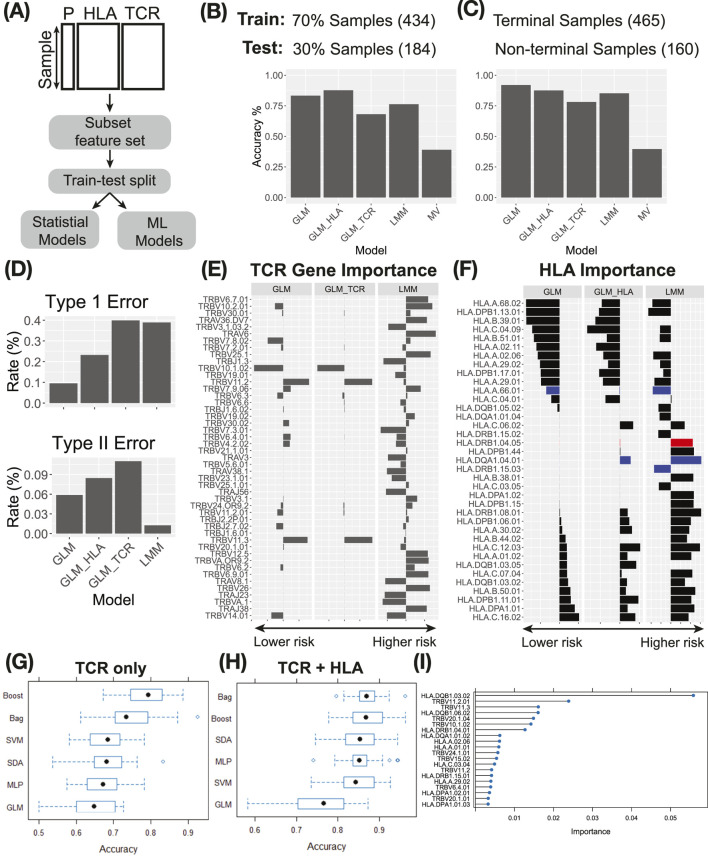
Statistical modeling and machine learning for the prediction of future T1D status using TCR gene abundance and immunogenetics as features. **(A)** Phenotypic (P), HLA allele identity (HLA) and scaled TCR gene abundance (TCR) were concatenated for each sample and split into training and test sets. GLMs were developed for the full dataset or a subset masking the HLA information (GLM_TCR) or TCR information (GLM_HLA). The LMM and MV models were trained on the full datasets. Phenotypic data included patient age, sex, study, and location was included in every model. Accuracy of each model was calculated for **(B)** a 70–30 train test split or **(C)** a model of the terminal samples trained to predict future disease risk of non-terminal samples. **(D)** The rates of type I error (False positive Rate, Top) or type II error (False negative Rate, Bottom) are shown for each model. **(E)** The top 20 TCR gene coefficients are displayed for each model of the full dataset as relative importance values. **(F)** The top 20 coefficients or intercepts of HLA allele identity are also shown as relative importance values. High risk HLA alleles are colored red and protective alleles are colored blue. A total of 5 machine learning models were trained using a 70–30 train-test split of the full dataset (TCR + HLA) or the normalized TCR gene abundance (TCR only). Accuracy of cross fold validations are reported for **(G)** TCR only or **(H)** TCR+HLA trained models. **(I)** Relative feature importance of the top 20 features from the random forest “Boost” model trained on the TCR+HLA dataset is displayed using SHAP values.

The current approaches to predicting the likelihood of developing disease suffer from high error rates. To address this, we evaluated the ability for TCR gene abundance and HLA allele identity to improve accuracy of prediction and reduce type I and type II error. TCR and HLA features were used to develop statistical models that predicted disease status (Figure 3A; Supplementary Figures S5A,B). A train-test split of 70–30 was employed to reduce overfitting of the models (Supplementary Figure S5C). The GLM trained on both TCR and HLA data outperforms a GLM trained only on TCR data (Figure 3B). The LMM shows comparable accuracy to the GLM but does not use any HLA identities as coefficients to the prediction thereby controlling for the overall effect of genetics. Overall, models built to predict just from HLA status showed consistent accuracy (80%) similar to previous reports (Sharp et al., 2019), however we recognize that the models likely contain biases based on unequal sampling of genetic backgrounds and incomplete genetic information from the original studies (Supplementary Figure S2).

The limitations in current strategies of early detection demonstrate that we need more accurate predictive measures at younger ages before disease pathogenesis begins. By training models with terminal measurements and predicting on non-terminal measurements, we increased GLM accuracy to 92% and achieved type I and II error less than 10% (Figures 3C,D).

The coefficients for TCR gene abundance show the relative importance of each TCR gene in determining risk. Abundance of genes such as TRBV11-3, TRBV11-2, TRBV10-1 and TRBV7-3 were highly important across models (Figure 3E). The intercepts and coefficients of HLA alleles used very few of the known risk or protective alleles in deciding future disease status (Figure 3F).

We also trained 5 machine learning models to predict future disease status using a 70–30 train test split of HLA, TCR, and combined features. As expected, using both TCR and HLA features improved model accuracy and AUC (Figures 3G,H; Supplementary Figure S5C). TRVB11-3, TRBV11-2, and TRBV10-1 importance in the random forest models measured through SHAP values and feature selection were consistent with the important TCR genes identified in the statistical models (Figure 3I; Supplementary Figure S6).

### 3.4 Population level RNA-seq data capture allele specific TCR gene dynamics

**FIGURE 4:**
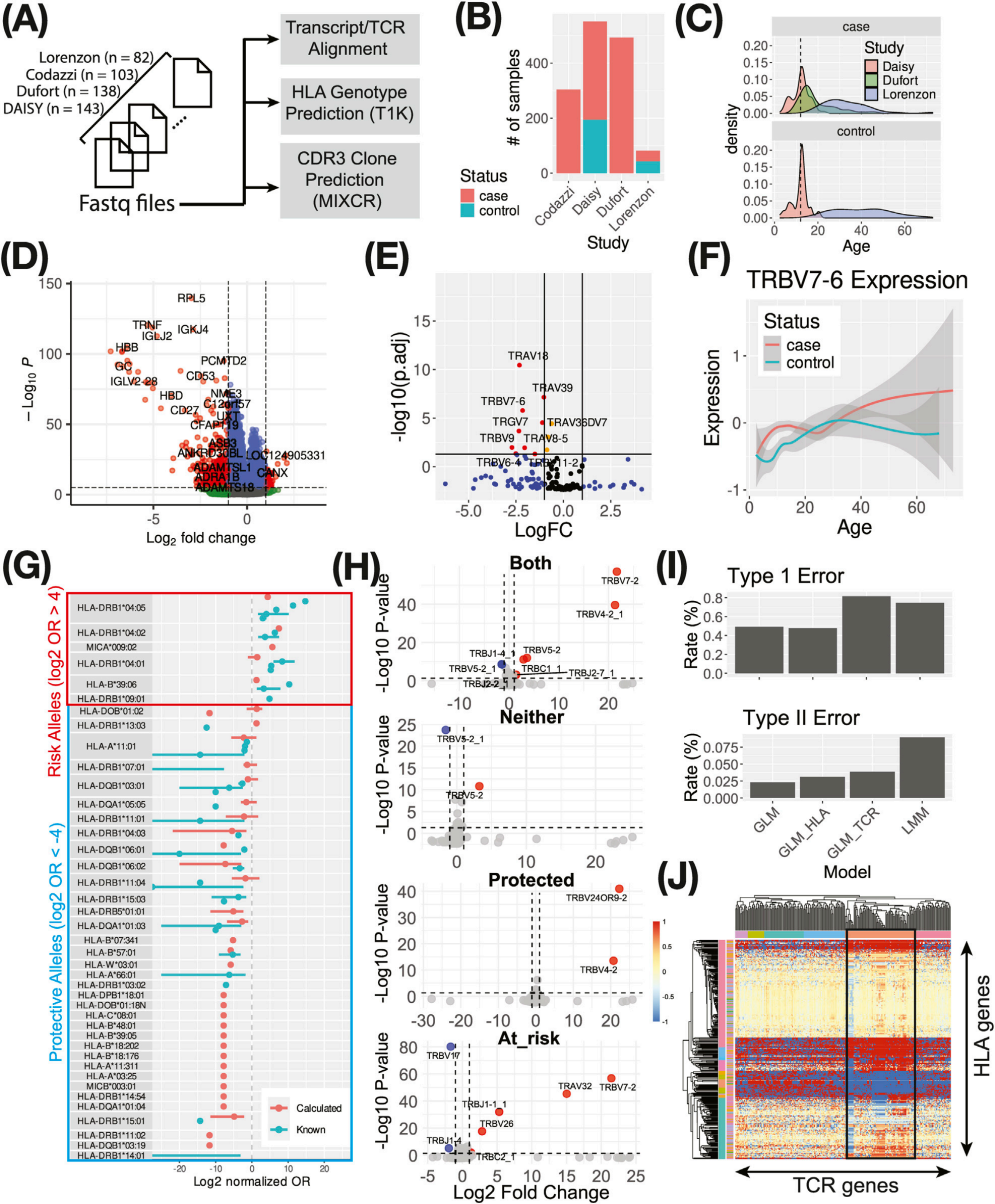
TCR prediction of diabetic status from bulk RNA seq data. **(A)** FASTQ files from 4 studies representing 466 patients were downloaded from the SRA database, trimmed, aligned to the human genome. **(B)** In total, 1,430 samples were run through the pipeline, with 1,193 classified as “case” and 237 classified as “control” based on reported phenotypes. **(C)** Density plot shows the age distribution of patients across studies. Codazzi samples reported no age measurement and Dufort samples only contain diabetic patients. **(D)** The result of differential gene expression (DEG) using DEseq2 is shown in volcano plot comparing “case” patients to “control” patients across studies. **(E)** Differential abundance of TCR genes was calculated and adjusted using FDR. TCR genes with absolute log fold change greater than 1 and adjusted p-value less than 0.05 are colored red and labeled. **(F)** The expression of TRBV7-6 is shown across ages for all samples. Lines represent the smoothed expression values calculated using a loess curve and grey regions represent the standard error of the interpolated mean. Lines are colored based on disease status. **(G)** Genotypes for HLA, MIC, and TAP alleles were predicted using the T1K program (Song et al., 2023). Detected alleles with Log2 Normalized OR >4 (red), are reported in comparison to previously known OR values. **(H)** Differential abundance of TCR genes was calculated for each allelic risk level and plotted as separate volcano plots. Genes are labeled if adjusted p-value is less than 0.05. Significant genes are colored red if log2 fold change is greater than 1 or blue if less than −1. **(I)** Statistical models were developed for features derived from bulk RNA-seq data as described before. Type I error (False positives, Top) and type II error (False negatives, Bottom) is reported for the full data model. **(J)** The correlation between TCR and HLA features was calculated using probabilities from the full data model. Pearson correlations are plotted as intensity values on a heatmap with TCR genes as columns and HLA genes alleles as rows. Rows are annotated by HLA gene (right) or cluster (left) and columns are annotated by TCR gene clusters. TCR gene cluster 3 is outlined with a black box.

TCR sequencing data for human T1D samples are less abundant and accessible than other sequencing modalities like bulk RNA-seq. Previous efforts have demonstrated that bulk RNA-seq data can be used to acquire the predictive features we identified including CDR3 clonal representation, TCR gene abundance, and HLA genotype (Bolotin et al., 2017; Chen et al., 2020; Song et al., 2023). We downloaded 1,430 whole blood RNA sequencing files from 4 different studies encompassing 373 patients with T1D and 93 healthy controls to determine if they contained information predictive of disease risk (Figures 4A,B; Supplementary Tables S8,S9) (Dufort et al., 2019; Carry et al., 2022; Codazzi et al., 2025; Valentim, 2025). Study ages ranged from 2 to 73 with the average patient having 3 RNA seq measurements at different ages of disease development (Figure 4C; Supplementary Figure S7). Normalized gene expression did not differentiate patients by disease status (Supplementary Figures S8A,B). While we did find 96 genes differentially expressed, few were involved in immune responses to T1D (Figure 4D; Supplementary Figure S8C; Supplementary Table S10). We did not observe overlap with contemporary analyses of T1D whole blood RNA seq samples (Suomi et al., 2023).

We used the MIXCR program to predict CDR3 sequences from raw fastq files (Supplementary Figures S8D,E). Average clonal recovery of unique CDR3 was around 200 per sample, which is low in comparison to the 10,000 on average CDR3 identified among TCR profiling samples. This is comparable to other reports of CDR3 recovery in RNA-seq datasets (Brown et al., 2015; Chen et al., 2020; Menda Dabran et al., 2025), but we observed no overlapping CDR3 across all samples.

By aligning RNA reads to transcript files we were able to recover expression of 243 TCR genes (averaging 106 per sample). Using the negative binomial model of normalization developed for single cell data (SCT) (Hafemeister and Satija, 2019), we were able to remove the effects of study and sequencing depth on TCR gene expression (Supplementary Figures S8F,G). We found that 9 TCR genes, including TRBV11-2 and TRBV7-6, were significantly decreased in individuals with T1D (Figure 4E; Supplementary Table S11). This was opposite to the trends observed in TCR profiling data and we determined it was mostly due to timing of measurement (Figure 4F; Supplementary Figure S8H). There was only 1 gene differentially abundant in the non-terminal patients which was TRBV23or9-2.

Next we used the T1K program to predict patient HLA genotypes from bulk RNA-seq files (Song et al., 2023). On average we recovered 33 complete alleles per sample, including MHC class I, class II, MIC and TAP loci (Supplementary Figure S9A). Per patient accuracy across sequencing measurements was 99.95%. This complete and accurate accounting of immunogenetic background in each patient allowed us to evaluate the relative risk of different loci. We found high correlation with previously identified risk alleles and identified previously unconsidered alleles that increased risk of disease like HLA-B*08:01 and MICA*027:01 (Supplementary Figure S10; Supplementary Table S12). We set a cutoff log2 normalized OR of 4 and -4 to classify allelic risk level which grouped 397 samples as Protected and 364 samples as At-risk based on allelic risk (Figure 4G; Supplementary Figure S9B). Allelic risk level had a large effect on differential TCR expression similar to the TCR profiling data (Figure 4H; Supplementary Table S13).

We developed similar statistical models as before for disease development. We observed accuracies in disease status prediction consistent with previously developed models (Supplementary Figure S9C). Rate of type I errors in the non-terminal samples increased to roughly 50% but type II error rates remained relatively the same (Figure 4I). Correlation between GLM TCR gene coefficients and allele identity showed a cluster of TCR genes with higher HLA allele dependence (Figure 4J). The results of these models indicate that future models will need to be built on larger datasets that reduce the class imbalance and have a more normal distribution of HLA alleles.

### 3.5 TCR specific T cells show disease relevant dysfunction

**FIGURE 5:**
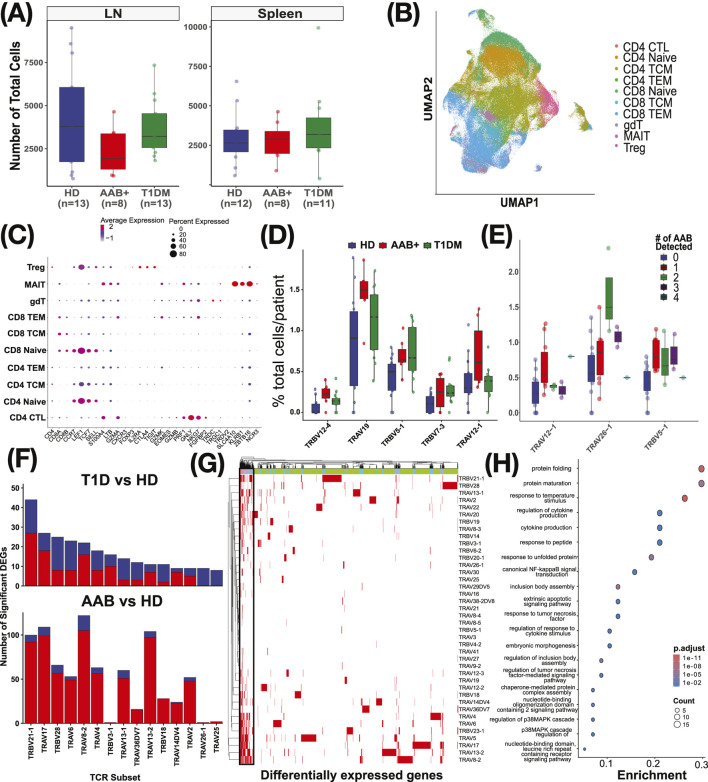
scRNA-seq of T cells from diabetic populations shows the effect of TCR gene usage on T cell function. **(A)** Total number of T cells recovered across clinical groups (AAB+, HD, and T1DM) stratified by tissue. **(B)** UMAP visualization of Harmony-integrated T cells from human spleen and lymph node (LN) samples. Cells are colored by T-cell sub type. **(C)** Dot-plot of marker-gene expression used to validate the biological consistency of annotated T-cell subsets in the integrated atlas. Dot size indicates the percentage of cells expressing each marker, and color indicates the average scaled expression. Donor-level TCR V-gene usage stratified by **(D)** disease status and **(E)** number of AAB detection. Displayed TCR genes were selected by significant difference in Kruskal-Wallis test. **(F)** Number of differentially expressed genes within each TCR gene containing cluster based on direction of comparative test. **(G)** Heatmap showing the shared detection of differentially expressed genes across TCR gene clusters in the T1D vs. HD comparison. DEGs common across most TCR gene clusters are outlined in a black box. **(H)** GSEA of common DEGs.

We have identified TCR genes whose abundance among circulating cells correlates with disease risk. Past the correlation with antigen targeting, we have no information as to why they would change based on disease status. To determine why T cells containing certain TCR genes may be differentially abundant leading up to disease, we analyzed a human single cell RNA-seq dataset from spleen and lymph node samples in 35 clinical T1D, single AAB+ pre-diabetic, and healthy donor (HD) subjects (Fasolino et al., 2022). In total, 220,338 T cells were isolated at an average of 6,295 cells per donor (Figure 5A; Supplementary Figure S11). T cell cluster annotations were confirmed with common markers and were found roughly equally represented in patients regardless of disease status (Figures 5B,C; Supplementary Figure S12).

At least 202 TCR genes were found expressed in 35,522 cells, with 31,630 cells found containing one V, D, or J TCRαβ or TCRγδ gene and 4,387 carrying multiple TCR genes (Supplementary Figure S13). We detected an average of 531 cells with at least one TCR gene in each individual, irrespective of disease status (Supplementary Figure S14A). TCR gene diversity within each sample did increase with total cell counts and higher sequencing depth (Supplementary Figure S14B).

TCR gene usage, the number of T cells with a specific TCR gene detected per sample, is analogous to TCR abundance and expression. T cells containing TRBV12-4, TRAV19, TRBV5-1, TRBV7-2, and TRAV12-1 were more abundant in the AAB+ individuals than individuals with clinical disease (Figure 5D). T cells containing TRAV26-1, TRAV12-1 and TRBV5-1 had a positive relationship with the number of AAB detected per individual (Figure 5E). We observed consistent changes in the usage of TCR genes in cells of different cell types, particularly for TRBV5-1 which is found more abundant in CD4^+^ memory cells, CD8^+^ memory cells, and CD4^+^ Tregs (Supplementary Figure S14C).

If the expression of a TCR gene changes its function, we expect disease relevant TCR gene clusters to have the most DEG in comparisons between healthy cells. TRBV21-1 containing T cells had the most DEG in T1D individuals, upregulating genes related to T cell activation alongside TRBV8-2 containing cells (Figure 5F; Supplementary Figure S14D; Supplementary Table S14). Conversely, we observed downregulation of T cell proliferation genes in TRAV26-1 and TRAV30 containing cells and cytotoxicity genes in TRBV3-1 containing cells. Genes most commonly differentially expressed across important TCR gene clusters were most involved in cytokine production and immune response (Figures 5G,H).

### 3.6 TCR gene usage predicts disease status based on polygenic risk

**FIGURE 6:**
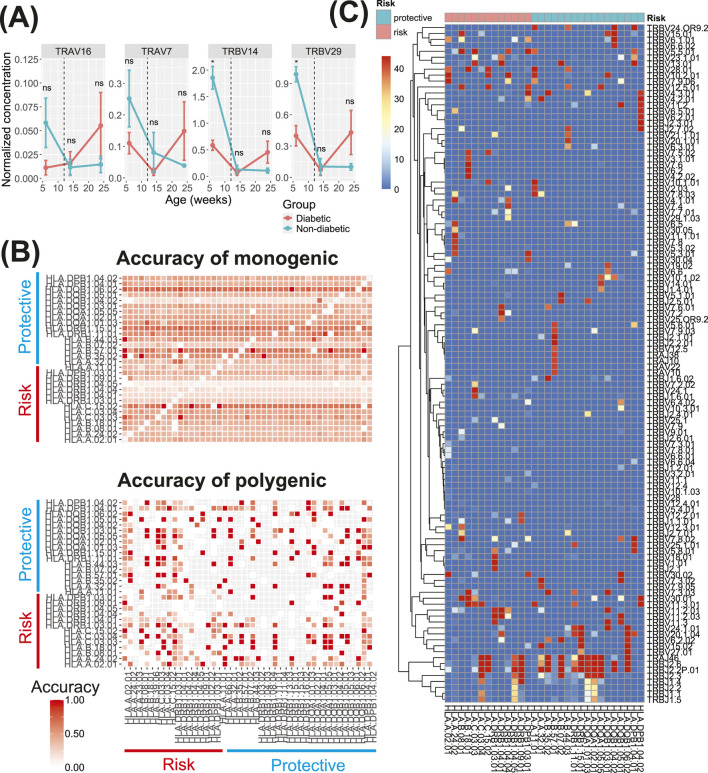
Evaluation of TCR gene markers in detecting future disease risk and marker selection in diverse populations. **(A)** The TCR genes; trav16n, trav7, trbv14, and trbv29 were quantified from circulating NOD mouse T cells at multiple time points with 5 replicates per disease status/time treatment. *p < 0.05 using a two-tailed t-test. **(B)** Independent GLMs were developed for patients based on polygenic combinations of risk and protective alleles. Accuracy values of model predictions are visualized using color values in a heatmap. Heatmaps represent accuracy (top) when one allele is present (y-axis) but the other is not (x-axis) or (bottom) when both alleles are present in an individual. **(C)** Cumulative importance of TCR genes per allele across polygenic models. A higher value (color intensity) represents the number of times each TCR gene was in the top 10 most important TCR genes. The disease risk for each HLA allele based on previously known OR is annotated for each column at the top.

To validate if changes in blood TCR gene abundance correlate and are predictive of disease progression, we performed dPCR on TCR genes in NOD mouse PBMC. We chose to measure the mouse TCR genes trav16n and trbv29, which we previously identified as predictive of disease risk (Islam et al., 2025). We also measured trbv14 and trav7 which are the mouse homologs of TRBV11-2 and TRAV12-1 in humans respectively. All 4 genes showed high expression at early (6 weeks) time points prior to the starting point of disease progression (12 weeks) (Figure 6A).

While mouse immunogenetics are homogenous, humans are polygenic for multiple risk alleles. These alleles affect TCR gene importance as we shown earlier and would make selection of markers for clinical assays more difficult. To determine TCR gene markers based on genetic background, we developed multiple GLM models for each polygenic combination of risk and protective alleles of the TCR profiling data. High accuracies were observed for specific combinations of alleles (Figure 6B). A majority of allele combinations were too sparsely sampled to develop predictive models. Based on cumulative polygenic risk, TCR importance varies, with classically identified TCR genes such as TRBV11-2 and TRBV11-3 showing high importance for predicting disease status in individuals with risk alleles, such as the DR3/4 variants (Figure 6C). Such results can be a reference for future development of molecular tests but would need greater diversity in patient samples to accurately determine marker effectiveness.

In total, this study identified 191 putative TCR genes that may be predictive of future disease development based on changes in expression within circulating blood populations (Supplementary Figure S15). We looked at the concordance between identified disease risk TCR genes and previously known TCR clones that are specific for T1D related antigens (Table 1) (Nakayama and Michels, 2021). Important TCR genes like TRBV11-2, TRBV5-1, and TRBV20-1 were commonly found in insulin specific TCR clones. While we identified that these genes could predict disease status, further investigation will be needed to better understand their interaction with genetics, lifestyle, and environmental factors.

**TABLE 1:** Identified important TCR genes compared to known epitope specific T1D clones. Data of known TCR gene usage was compiled from (Nakayama and Michels, 2021).

​	TCR sequencing		​	​	Bulk RNA sequencing		Single cell
Gene	Clones	Differential abundance	Statistical models	ML models	Differential abundance	Allele dependent	Gene usage
GAD65	BRI4.13 & BRI164	TRBJ1-6	TRBV5-1	TRBJ1-6	TRBJ1-1	TRBJ1-1 & TRBV5-1	TRAV19 & TRBV5-1
IGRP	T1D2-1&2 & T1D4-3&4 & Clone_7 & Clone_32 & Clone_16/17 & Clone_22/27	TRBJ2-3 & TRBJ2-4 & TRBV6-6 & TRBV6-2/6-3 & TRBV20-1 & TRBV3-1	TRBJ2-1 & TRBV6-6 & TRBV5-1 & TRBV6-2/6-3 & TRBV20-1	TRBJ2-7 & TRBV20-1	TRAV2 & TRBJ2-7 & TRBV3-1 & TRAJ53	TRAV2 & TRBJ2-7 & TRBJ2-4 & TRBJ1-2 & TRBV5-1	TRAV2 & TRAV12-1 & TRAV25 & TRBV5-1 & TRBV3-1
PPI	23.G8 & SD52.c1 & 95.A9-1 & 6.H11 & SD32.5 & 23.F7 & 55.B3 & 55.C10 & 19.A4 & GSE.8E3 & 6.G4 & 56.B1 & 53.A4-1 & 23.G6 & 1.00E+06 & 23.F9 & 19.A1 & 2.00E+06 & 20.F1 & 22.A10 & 1.C8 & 1.F3 & 96.F5 & 23.H5 & 1.B10-1 & 1.F1 & 23.C12 & 93.D1 & 10.C6-1 & 28.D3 & 2.80E+07 & 20.G1 & 96.B4 & 86.C1 & 84.D9 & 2.80E+12 & 1.E9-1 & 86.G3-2 & 54.F1	TRBJ1-6 & TRBJ2-3 & TRBJ2-6 & TRBJ2-4 & TRBV24-1 & TRBV11-2 & TRBV20-1 & TRBV4-1 & TRBV7-9 & TRBV29-1 & TRBV12-4 & TRBV6-1 & TRBV4-2/4-3 & TRBV6-2/6-3 & TRBV7-2	TRBJ2-1 & TRBV11-2 & TRBV20-1 & TRBV7-9 & TRBV5-1 & TRBV6-1 & TRBV6-2/6-3	TRBJ1-6 & TRBJ2-7 & TRBV24-1 & TRBV11-2 & TRBV20-1	TRAV39 & TRAV2 & TRAV13-1 & TRAV8-1 & TRAV8-3 & TRAV1-2 & TRAV3 & TRBJ2-2 & TRBJ1-1 & TRBJ2-7 & TRBJ1-4 & TRBV11-2 & TRBV4-1 & TRBV7-9 & TRBV12-4 & TRBV9 & TRBV6-1 & TRBV4-2/4-3 & TRBV2 & TRBV7-2 & TRAJ52	TRAV2 & TRAV3 & TRBJ2-2 & TRBJ1-1 & TRBJ2-7 & TRBJ1-4 & TRBJ2-4 & TRBJ1-2 & TRBV4-1 & TRBV5-1 & TRBV28 & TRBV10-2 & TRBV9 & TRBV6-1 & TRBV4-2/4-3 & TRBV2 & TRBV7-2	TRAV4 & TRAV26-1 & TRAV8-2 & TRAV2 & TRAV13-1 & TRAV5 & TRAV19 & TRAV1-2 & TRAV3 & TRBV18 & TRBV5-1 & TRBV12-4 & TRBV28 & TRBV2
Insulin_A	Mi.1 & Ba.14 & Ba.11	TRBJ2-3 & TRBV29-1	TRBV5-1	​	TRAV8-3 & TRAV39 & TRAJ52	TRBV5-1	TRBV5-1
Proinsulin	B3.3 & K4.4/K6.4 & A4.13 & A1.1 & A1.2 & A2.4 & B3.1 & K3.2/K9.5 & K6.2 & K9.6 & D1.1/D1.4 & T6.1 & T6.6 & T17.1 & H3.3/H6.4 & H3.7/H7.4/H8.5 & H11.5 & E2.3 & E2.5 & A1.9 & A6.15/A5.8 & A2.13 & A5.5 & ET650-2 & ET650-4 & ET672-1	TRBJ2-3 & TRBJ2-4 & TRBJ1-6 & TRBV7-8 & TRBV20-1 & TRBV7-2 & TRBV3-2 & TRBV4-2 &	TRBJ2-1 & TRBV7-8 & TRBV20-1 & TRBV5-1 & TRBV3-2 & TRBV5-4 &	TRBJ2-7 & TRBJ1-6 & TRBV20-1 &	TRAV3 & TRAV13-1 & TRBJ1-1 & TRBJ2-7 & TRBJ2-2 & TRBJ1-4 & TRBV7-2 & TRBV4-2 & TRBV9 & TRAJ9 & TRAJ5	TRAV3 & TRBJ1-1 & TRBJ2-7 & TRBJ2-4 & TRBJ2-2 & TRBJ1-4 & TRBJ1-2 & TRBJ1-3 & TRBV5-1 & TRBV7-2 & TRBV4-2 & TRBV9 & TRBV5-4 & & TRAJ5	TRAV17 & TRAV25 & TRAV19 & TRAV12-1 & TRAV3 & TRAV8-2/8–4 & TRAV26-1 & TRAV13-1 & TRBV18 & TRBV5-1 &
Insulin_B	Clone_5 & GSE.6H9 & GSE.20D11 & T1D#3_C8 & T1D#3_C10	TRBV11-2 & TRBV7-2 & TRBV4-1	TRBJ2-1 & TRBV11-2 & TRBV5-1	TRBV11-2	TRBJ2-2 & TRBV11-2 & TRBV7-2 & TRBV2 & TRBV4-1	TRBJ2-2 & TRBV7-2 & TRBV2 & TRBV5-1 & TRBV4-1	TRAV26-1 & TRAV17 & TRBV2 & TRBV5-1
INS-DRiP	Clone#1 & Clone#2 & 1.C1 & 96.A9	TRBV6-1 & TRBV12-3/12-4	TRBV6-1	​	TRBJ2-2 & TRBJ1-1 & TRBV6-1	TRBJ1-2 & TRBJ2-2 & TRBJ1-1 & TRBV6-1	TRAV12-1
ZNT8	D222D_Clones_2 & D010R_clone_1E2 & D010R_clone_1D3 & D267T_33B8 & D349D_178B9 & D351D_188D3	TRBJ2-3 & TRBV6-1 & TRBV11-2	TRBV6-1 & TRBV11-2	TRBJ2-7 & TRBV11-2	TRBJ2-2 & TRBJ2-7 & TRBJ1-1 & TRBV6-1 & TRBV11-2 & TRAJ9	TRBJ2-2 & TRBJ2-7 & TRBJ1-3 & TRBJ1-1 & TRBV6-1	TRAV17 & TRAV25 & TRAV19

## 4 Discussion

In this study, we have explored how T cell specific sequencing data predicts future clinical diabetes in populations of humans. The utility of T cell biomarkers has been previously explored for their use in T1D disease prediction (Ahmed et al., 2019; Nakayama and Michels, 2021; Yang et al., 2022). In comparison to AAB based approaches, epitope specificity assays for T cells have been less viable in the clinical setting. Activation assays are not standardized due to the effect of different genetics on antigen presentation and T cell response *in vitro* (Scotto et al., 2012). Newer pMHC multimer based binding assays can be measured through flow-cytometry but face similar barriers of HLA allele and epitope selection (Cernea and Herold, 2010). For these reasons, the development of a T cell assay for epitope specific responses equivalent to B cell based assays has proven impossible or too expensive to implement. Attention has been given to TCR repertoire based approaches, however as we point out in this paper, overlap of specific CDR3 is so low (generally <1% between two individuals) that finding a common CDR3 marker is like finding a needle in a proverbial haystack.

However, there may be better ways to develop TCR based assays. By 2003 it had been confirmed that oligoclonal TCR populations were overrepresented in autoimmune responses, but it was suggested that there were no TRBV families associated with this effect (Hodges et al., 2003). As we demonstrate in this paper, TCR gene usage in circulating cell populations is differentially enriched in the disease state and are predictive of future disease onset at early time points. TCR genes marked as disease predictive in our models (including TRBV11-2, TRBV7-3, TRBV10-2, TRBV24-1, and TRBV5-1) are constituents of known diabetic TCR clones targeting Preproinsulin, insulin, Znt8, IGRP, and IAPP2 (Eerligh et al., 2011; Unger et al., 2012; Pathiraja et al., 2014; Babad et al., 2015; Tan et al., 2017; Yeh et al., 2017; Culina et al., 2018; So et al., 2018; Anderson et al., 2021; Landry et al., 2021; Tran et al., 2021). As we show using preclinical models, these genes can be measured directly from blood samples using dPCR or qPCR based assays and demonstrate similar disease dynamics to the human sequencing data. Other studies have demonstrated that similar measurements of TCR gene usage can be made using flow cytometry which is a more sensitive report at the cellular level (Okamura et al., 2022). Such a technique can also be paired with markers of T cell function, which is interesting considering we found that TCR gene usage was correlated with cell type differentiation (i.e., TRBV5-1 being more enriched in diseased Treg cells). A flow sorting based approach could also be used to isolate TCR gene specific T cells to further characterize *in vitro* differences such as cytokine production and response.

The importance of our main finding is that the utility of each marker in predicting disease is variable based on genetic background of HLA alleles (and possibly other genetic markers). While there doesn’t seem to be a general marker that is consistent across all genotypes, we did find that creating polygenic models allows marker selection at the individual level. Both TCR gene and HLA allele features can easily be captured in a clinical setting using a single cheap RNA-seq based test. Further development into TCR gene biomarkers should be prioritized increasing the variability of sample populations in developing an established clinical doctrine using RNA-seq based technologies. Continued efforts to develop such assays in autoimmune disease can also contribute to and take lessons from the current development of TCR based assays for cancer detection (Ni et al., 2022; Li et al., 2025).

## Data Availability

The original contributions presented in the study are included in the article/Supplementary Material, further inquiries can be directed to the corresponding author.

## Supplementary material

SUPPLEMENTARY FIGURE S1 TCR profiling data by patient age and study.

SUPPLEMENTARY FIGURE S2 Bar plot showing the number of HLA alleles detected per study and allele class.

SUPPLEMENTARY FIGURE S3 Characterization of CDR3 sequences from TCR profiling studies. **(A)** Boxplot showing the distribution of unique CDR3 sequences per sample in each study. **(B)** The number of unique CDR3 sequences per sample is plotted against the total number of detected CDR3 reads. Each point representing a sample is colored by study. **(C)** A barplot representing the percent of CDR3 clones that are detected in multiple patients. **(D)** 100,000 unique CDR3 sequences were sampled to calculate the percent of total samples they were found in.

SUPPLEMENTARY FIGURE S4 Processing and analysis of CDR3 clustering and CDR3 genes from TCR profiling data. **(A)** Average number of patients detected based on cluster size. **(B)** The sum of normalized aggregate cluster abundance is plotted for each patient. A minimum cutoff of 10 (dashed line) was set to remove outliers for cluster abundance. **(C)** Mean normalized TCR abundance was plotted per sample. **(D)** Mean expression of differentially abundant TCR genes were plotted as bar plots which color representing disease status. Black bars represent standard deviation. *p < 0.05 in Welch’s t-test. **(E)** Venn diagram shows the number of differentially abundant TCR genes shared by tests made on subsets of patients with different allelic risk levels.

SUPPLEMENTARY FIGURE S5 Quality metrics for statistical and machine learning models. Schematic of statistical models used in the study. **(A)** Feature dataset with the number of rows equal to the number of samples (n) was used to develop statistical models. The identity matrix of each detected HLA (k) and the scaled abundance values of each TCR (m) were used as coefficients of GLM models. The LMM models developed a different intercept applied based on detected alleles and the MV model developed a unique set of TCR coefficients for each Allele. Solid black lines represent the model formed from the actual coefficients. Representative points are colored based on allele identity, demonstrating the allele effect. **(B)** Quality metrics measured per HLA from each model and feature subset. Plots are organized by row based on what metric was being measured. Plots are organized by column based on which subset of data were used to develop each set of models. The training and testing data are represented by in parenthesis and most models were trained using a random 70-30 train-test split, T-nT represented the terminal samples trained to predict the non-terminal samples and vice-versa for nT-T. Points represent the measurement for a subset of patients that are either all test patients (green), “high-risk” alleles (red), “highly-protective” alleles (blue), or any other HLA allele (black). **(C)** Training and testing accuracy of the full data statistical models are represented to show a lack of overfitting to the training set. **(D)** Receiver-operator curves (ROC) show the sensitivity and specificity for machine learning models trained on TCR only or TCR+HLA feature sets. Models are compared on the same plot and represented with different colors. A dashed line in the center represents a 50-50 guess for the binary disease status prediction.

SUPPLEMENTARY FIGURE S6 Feature selection using recursive feature elimination (RFE) with two models Random Forest (left) and the Generalized Linear Model (right). RFE calculates accuracy associated with a random selection of features (mean reported on the y axis) of different sizes to show the number of features associated with highest prediction accuracy. The best accuracy mean is represented with a blue dot. The top 20 features from the best feature model were plotted by their overall importance (SHAP values) (bottom).

SUPPLEMENTARY FIGURE S7 RNA-seq data patients plotted by age. Connected lines indicate patient blood samples sequenced at multiple time points. Points and lines are colored by diabetic status.

SUPPLEMENTARY FIGURE S8 RNA and TCR gene expression in bulk RNA-seq files. **(A)** Boxplots showing expression of all protein coding genes based on raw counts (top) or vst normalized counts (bottom). **(B)** Principal component analysis for normalized counts in RNA seq files colored by disease status. Different point shapes represent the study the file came from. **(C)** Gene set enrichment analysis of DEG in case samples vs control. **(D)** Number of unique CDR3 detected in each sample using MIXCR with the “rna-seq” preset plotted by clonality of detected TCR. **(E)** Number of unique CDR3 detected in each sample plotted against the total number of reads from each RNA-seq file. **(F)** Average expression of TCR constant region genes per sample is plotted in red, with gray line representing the standard deviation. Mean is plotted from raw, vst normalized and SCT normalized values. **(G)** Sequencing depth in total read counts for each sample is plotted against mean raw TCR gene expression or mean SCT normalized TCR gene expression. Each sample is colored based on study. **(H)** Differentially expressed TCR genes were as an interpolated (mean) value across age. Loess smoothed curves are colored by disease status, and shaded region represents the standard error around the calculated value.

SUPPLEMENTARY FIGURE S9 Allele prediction and statistical models predicted from RNA-seq derived features. **(A)** Samples are plotted by sequencing depth against the number of HLA alleles detected per sample. Number of HLA alleles detected does not differentiate homozygous alleles in which only one allele is present versus prediction error where only one allele is detected. **(B)** The number of samples that are categorized by allele risk level based on a variable cutoff OR for risk and protection level. **(C)** Accuracy measurements for statistical models trained on different subsets of the feature set.

SUPPLEMENTARY FIGURE S10 Genetic risk factors for diabetic onset. OR was compiled from previous studies (blue) or calculated from patients in the study based on bulk RNA-seq predicted alleles (red). Error bars represent the 95% confidence interval of estimates. OR is log2 normalized then multiplied by 2 so that OR below 1 is represented as negative values.

SUPPLEMENTARY FIGURE S11 Cell count per donor between 2 different tissue sources.

SUPPLEMENTARY FIGURE S12 Average proportion of cells per condition and cell cluster to the total number of cells in each sample.

SUPPLEMENTARY FIGURE S13 The number of cells with detected TCRV,D, or J genes. Pipe plot (bottom-right) shows the number of cells in each single positive or multi-positive detection for V, D, or J genes in TCRα, β, γ, or δ clonotypes.

SUPPLEMENTARY FIGURE S14 Gene usage in single cell RNA-seq T cells. **(A)** T cells with a detected TCR gene per sample are plotted based on disease status. **(B)** V gene diversity was linearly associated with the total number of cells in each sample and the median number of UMIs detected per cell. **(C)** TCR genes that statistically differ in TCR gene usage are plotted per cell type. The proportion of total T cells per sample in each cell type are plotted on the y-axis. Statistical significance determined by p value < 0.05 on Kruskal-Wallace test for each TCR gene. **(D)** Gene set enrichment analysis of DEG in each TCR gene cluster for the comparison of T1D vs HD patients.

SUPPLEMENTARY FIGURE S15 mportant TCR genes in each analysis from the paper were plotted as a heatmap to visualize the TCR genes shared by multiple analyses. TCR genes that were important in at least one analysis are represented by red if they are represented and blue if they are not for each analysis method.
